# Circadian lifestyle determinants of immune checkpoint inhibitor efficacy

**DOI:** 10.3389/fonc.2023.1284089

**Published:** 2023-12-04

**Authors:** Bethan R. Hughes, Sadiq Shanaz, Seline Ismail-Sutton, Nicholas I. Wreglesworth, Christian P. Subbe, Pasquale F. Innominato

**Affiliations:** ^1^ Oncology Department, Ysbyty Gwynedd, Betsi Cadwaladr University Health Board, Bangor, United Kingdom; ^2^ School of Medical Sciences, Bangor University, Bangor, United Kingdom; ^3^ Department of Acute Medicine, Ysbyty Gwynedd, Bangor, United Kingdom; ^4^ Cancer Chronotherapy Team, Warwick Medical School, University of Warwick, Coventry, United Kingdom; ^5^ Research Unit ‘Chronotherapy, Cancers and Transplantation’, Faculty of Medicine, Paris-Saclay University, Villejuif, France

**Keywords:** cancer, immunotherapy, circadian, diet, exercise, light, lifestyle

## Abstract

Immune Checkpoint Inhibitors (ICI) have revolutionised cancer care in recent years. Despite a global improvement in the efficacy and tolerability of systemic anticancer treatments, a sizeable proportion of patients still do not benefit maximally from ICI. Extensive research has been undertaken to reveal the immune- and cancer-related mechanisms underlying resistance and response to ICI, yet more limited investigations have explored potentially modifiable lifestyle host factors and their impact on ICI efficacy and tolerability. Moreover, multiple trials have reported a marked and coherent effect of time-of-day ICI administration and patients’ outcomes. The biological circadian clock indeed temporally controls multiple aspects of the immune system, both directly and through mediation of timing of lifestyle actions, including food intake, physical exercise, exposure to bright light and sleep. These factors potentially modulate the immune response also through the microbiome, emerging as an important mediator of a patient’s immune system. Thus, this review will look at critically amalgamating the existing clinical and experimental evidence to postulate how modifiable lifestyle factors could be used to improve the outcomes of cancer patients on immunotherapy through appropriate and individualised entrainment of the circadian timing system and temporal orchestration of the immune system functions.

## Introduction

Discoveries in immunotherapy have revolutionised cancer treatment. In 2018, James Allison and Tasuku Honjo won the Nobel Prize in Medicine for their work investigating the proteins CTLA-4 and PD-1 (1). Found on T cells, these proteins acted as checkpoint molecules, moderating T cell activation and preventing over-activation. As T cells are also involved in immunosurveillance of cancer cells (2), tumours can exploit CTLA-4 and PD-1 expression to evade host immune response. Inhibiting these checkpoint molecules can therefore enhance the antitumour immune response (3).

The antibodies subsequently developed to target checkpoint molecules and block their function are referred to as immune checkpoint inhibitors (ICI) and are the most widely used form of immunotherapy in cancer clinics. Currently, ICI are licensed to treat a wide array of cancers, including melanoma, lung, head and neck, renal, mesothelioma, breast, oesophageal, gastric, colorectal, biliary tract and urothelial carcinomas (4).

However, primary or acquired resistance remains a problem even with ICI (5). In addition, over activation of T cells can endanger self-tolerance, with the unavoidable risk of developing potentially life-threatening autoimmune adverse effects even years following initial treatment (6, 7).

In patients unlikely to respond to ICI therapy, accurate prediction of efficacy and tolerability would allow clinicians to minimise adverse effects and delays to these inherently time-pressured treatment plans. For many reasons, including difficulties developing appropriate *in-vitro* assays, the determinants of ICI efficacy, tolerability, and deleterious interactions are not fully understood, but are generally appreciated to be multifactorial and likely involve both modifiable and non-modifiable factors.

Many biomarkers both from the original tumour and circulating cells have emerged as areas of research interest into the impact of ICI efficacy and/or tolerability (8, 9). Alongside these factors, recent reports have highlighted the relevance of the circadian timing system (10). In turn, the CTS function is influenced by a host of lifestyle factors (11, 12). Lifestyle factors are of particular interest to clinicians, as they allow outcomes to not only be predicted, but to be potentially manipulated as well. This aspect of non-pharmacological interventions in oncology is indeed rising growing interest recently (13, 14). In this review, we critically summarise existing evidence on key lifestyle factors of interest – diet, physical exercise, and bright light exposure – with regards to ICI efficacy, through CTS manipulation, and impact on the immune system and the microbiome. We then discuss how these factors all interact to form a complex web which, with further understanding, may be manipulated by the empowered patient in conjunction with clinicians and various specialised healthcare professionals to optimise response to cancer immunotherapy (15, 16).

## The circadian timing system

The human body has an inherent timekeeping ability. Its internal ‘clock’ is thought to have evolved thanks to the survival advantage conferred by the ability to predict bodily requirements and adapt accordingly (17, 18). Circadian (i.e., with a period of about 24 hours) rhythms reflect the nature of the world humans have evolved in – being that environmental properties change with time in a predictable pattern based on the Earth’s rotation. In humans, the CTS hierarchically involves a central pacemaker, the suprachiasmatic nucleus in the ventral hypothalamus, and peripheral oscillators, temporally coordinated by hormonal, neural and physiological cues (19). Timing within cells themselves involve transcription-translation feedback loops and post-translation modifications involving a set of core clock genes, which encode proteins with limited half-lives (17). The rhythmic oscillations in core clock genes coordinate, in a tissue specific function and directly and indirectly, circadian transcription of selected genes, which ultimately engender variation in cellular functions over the 24-hour period, including cancer- and immunity-related hallmarks (20–22).

Although the CTS does not require external input, it can be entrained using external stimuli, such as light exposure. Other stimuli which can entrain the CTS include feeding times, exercise, and social schedules (11, 12, 23–26). Consequentially, manipulation of exposure to rhythmic entraining cues can be used to enhance or shift the CTS function (27), with potential benefit for patients’ wellbeing (28).

Timekeeping behaviour is also important to the immune system. Intrinsic clocks have been demonstrated to be present in a number of innate immune cells, causing rhythmic gating of function as well as regulating temporal spatial abundance (29). Natural killer cell cytolytic activity was found to be suppressed in correlation with altered clock gene expression in rats experiencing a simulation of chronic shift-lag, which was also associated with increased lung tumour growth (30). Additionally, experimental disruption of host circadian rhythms has shown to create an immunosuppressive remodelling in the tumour microenvironment, promoting cancer-cell proliferation and metastatic spread (31, 32). Evidence of circadian rhythmicity has also been found in the adaptive immune system in regulating CD8^+^ T-cell and dendritic cell differentiation and trafficking, with implications in cancer immunotherapy (29, 33). Studies in night shift work in humans corroborate experimental evidence on a negative effect of circadian disruption on immune system physiology (34).

The circadian rhythmicity of the innate and adaptive immune system ensures proportionate responses to infections, whereas dysregulation presents acutely in an inflammatory cytokine syndrome or manifests long-term as chronic inflammatory conditions, with relevant therapeutic implications in oncology as well as in many other medical conditions and procedures (35, 36).

The taxonomic composition of the microbial ecosystem, principally but not solely in the gut, has been associated with the incidence and clinical course of many different diseases, as well as with response to specific treatments (37). The mechanisms involved, which can display circadian oscillations (38, 39), include direct vagal stimulation, inflammation processes, and production of cytokines and metabolites (38, 40). Of paramount interest here is the growing evidence of the impact of the gut microbiota on ICI efficacy (41). Alongside this, the CTS and microbiome have been demonstrated to have intertwined relationships illustrated best by research showing how in combination they can synchronize bi-directionally the body’s metabolic response to diet (42) as well as light (43), exercise (44) and socialisation (45). Thus, the gut microbiome, itself potentially modifiable through iatrogenic interventions (46) takes a pivotal role in the rhythmic interplay among malignant processes, metabolism and immunity (13, 38, 47–49). Indeed, as a developing theme, the gut microbiome has been demonstrated as having effects on innate immunity, adoptive immunity and intriguingly direct within the tumour microenvironment (50).

**Figure 1:**
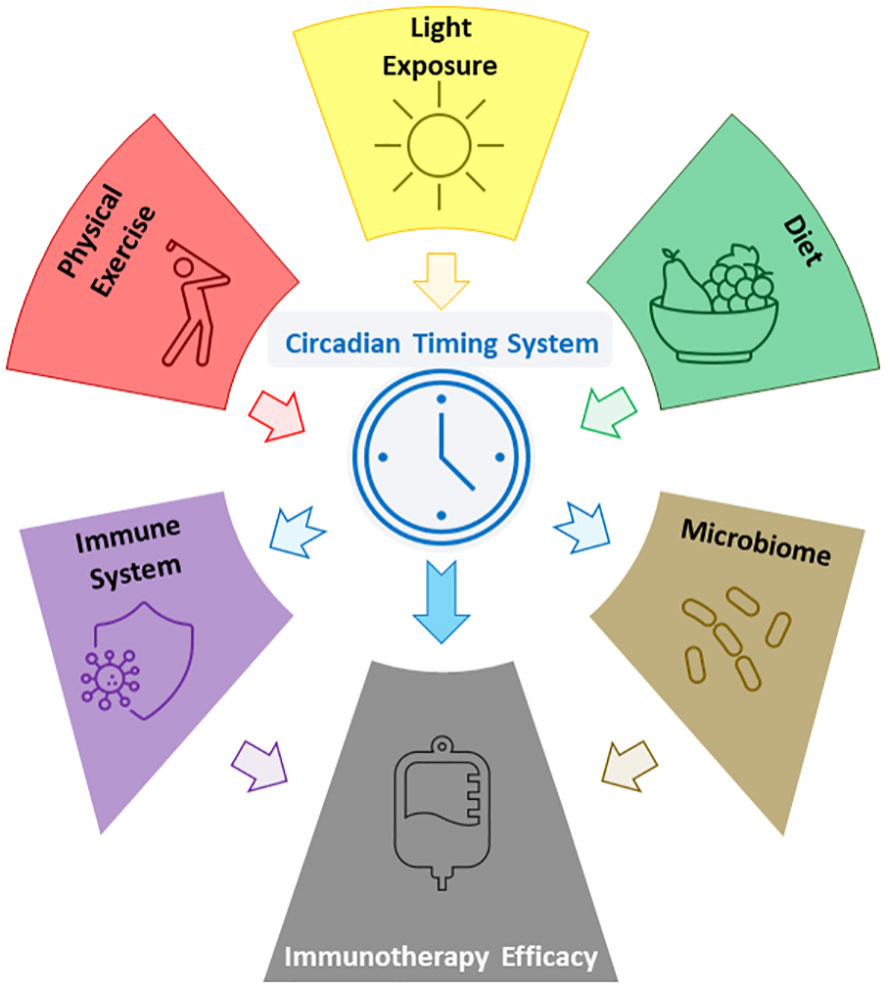
Figure showing some of the reported relations between lifestyle factors, the CTS, the microbiome, and ICI efficacy.

The link between the immune system and the CTS has been used to investigate potential ways of optimising response to various cancer treatments, including ICI. In particular, the time of day of ICI administration has been shown to be an independent prognostic factor for overall survival in several cancer types, with consistent findings disfavouring late afternoon administration (51). As the CTS can be entrained through modifications to lifestyle determinants, it therefore stands to reason that via the CTS certain lifestyle modifications could ultimately positively impact overall survival in cancer patients receiving ICI (**Figure 1**).

### Light exposure

Photic signals from the retina to the suprachiastmatic nucleus (SCN) encodes time of day information regarding the environmental surroundings (52). Photic signalling integrated in the SCN modify cellular and molecular activity of astrocytes, neurones and synchronises peripheral clock activity of organs (52, 53). The SCN interacts with peripheral clocks via inputs from the endocrine and adrenergic nervous system (54) resulting in activation of immune cells (55).

However, photic signalling information can be altered in cancer patients because of insufficient physiological bright light exposure during the day and experience of artificial light at night (ALAN). For example, ALAN affects both innate and adaptive immune function in invertebrates, birds, and rodents with robust pineal melatonin rhythm (43). Abnormal photic exposure not consistently encoding time-of-day information can affect innate and adaptive immune activity (44, 45, 56, 57), potentially impacting the efficacy of ICI. Although the impact of circadian photic schedule on ICI remains putative to date, in other conditions such as in psychiatry, light-based chronotherapy has shown positive therapeutic interactions with pharmacological and other behavioural interventions (58–60). Although prone to intrinsic constraints in the anatomical region of measured light exposure, contemporary digital tools allow for circadian evaluation of light exposure schedule and intensity, with potential cancer chronoimmunotherapeutic overtones (61, 62).

It is shown trafficking of immune cells are affected in a time of day dependent fashion (63, 64) Photic exposure entrains immune cell trafficking via the adrenergic nervous system (63, 64). No direct evidence exists to our knowledge on the impact of photic scheduling and entraining effects on outcomes of ICI, chemotherapy or other targeted therapies used in cancer. There are experimental and clinical data to support the impact of daytime bright light exposure and avoidance of artificial light at night, on both the immune and microbiome activity (43, 65–68). For instance, in the absence of light, the sympathetic nervous system triggers the pineal gland to produce melatonin, which synchronises SCN activity to peripheral clocks of immune cells (57, 69). Melatonin regulates innate, cellular and humoral responses of the immune system through modulating production of cytokines and oxidative stress (57). Additionally, melatonin acts as an immunostimulant under basal or immunosuppressed conditions, providing more effective early immune response against external stressors, such as viruses or parasites (70). However, in transient or chronically exacerbated immune response states, melatonin exerts negative regulation and can be regarded as an anti-inflammatory molecule (71).

The activity of the immune system is also influenced by Vitamin D, which is produced because of sunlight’s effect on keratinocytes (72, 73). Furthermore, Vitamin D modulates the gut microbiome and its metabolic activity, which is shown to be influenced by ALAN (69, 74). Thus, Vitamin D deficiency is linked with inflammatory bowel disease (75), obesity (76), diabetes (77), pro-inflammatory cytokine production (78), intestinal barrier disturbance (79), gut dysbiosis (80) and immune-mediated disease (81).

### Physical exercise

Physical exercise has been shown to strongly entrain the human circadian timing system, particularly through its effects on skeletal muscle and the cardiovascular system (82, 83). Furthermore, peripheral clocks within cardiovascular cells are key for modulating endothelial function, vasodilation, resistance, blood pressure, heart rate and several other key functions (84). Aerobic exercise induces neuroendocrine changes including increased production and release of melatonin and lower cortisol levels at night. This allows resynchronisation of the circadian clock, resulting in better sleep quality, and lower blood pressure and heart rate (82).

Entrainment of the circadian rhythm via exercise occurs even through low-intensity exercise and may be partly driven by changes in body temperature during physical activity (85). The degree to which physical exercise increases the body temperature is also dependent on the circadian phase, being larger in the rest phase than in the active phase (84). Moreover, combining photic cues, and non-photic exercise cues, has been shown to result in entrainment of the human clock at a faster rate than those with limited exercise (82).

Altogether, appropriate physical exercise in terms of timing, intensity, duration and type, adapted to the individual constraints of cancer patients, can be exploited to entrain the CTS and increase the robustness of circadian rhythms. Mobile health devices can lend useful tools to implement tailored circadian-based exercise schedules, even in cancer patients on ICI (16, 86, 87).

Although no direct evidence on the impact of physical exercise on outcomes on ICI is available to date, its impact on both the immune system and the microbiome supports its individualised manipulation to try to increase circadian-based ICI efficacy. Indeed, physical exercise has several immunomodulatory effects, including immune cell mobilisation in the blood, particularly PD-1+ CD8+ T cells redirected to peripheral tissues, which are crucial for host defence against tumours (88, 89).

An additional study analysing mice with pancreatic cancer demonstrated an improved responsiveness to immunotherapy in mice that exercised regularly, compared to those that did not. Mice who had regular exercise also had a greater antitumor response and an increased volume and influx to tumours of NK and CD8+ T cells (90).

Moreover, regular exercise influences the gut/brain axis, leading to an anti-inflammatory, immunoregulatory state and enriched gut microflora diversity (91). Indeed, multiple factors have been associated with intestinal dysbiosis in cancer patients (92), and physical exercise, alongside diet, could be a potentially modifiable element to ameliorate the gut microbiome in order to maximise benefit from ICI.

### Diet

Our eating habits generally follow a broad pattern that repeats every 24 hours. This pattern will vary from person to person and culture to culture, however commonly it may include three meals of various composition at a similar time each day. Interactions between the CTS and diet can therefore be divided into those relating to meal timing and those related to meal composition. Evidence suggests both these factors interact with the CTS (25, 93).

Food is one of the main synchronisers of the peripheral clocks (94). Both meal timing and meal composition can disrupt and re-programme the CTS by altering clock gene expression, causing reorganisation of liver metabolic pathways and altered pancreatic insulin secretion (95, 96). Thus, with regards to circadian entrainment, both timing, including fasting duration, and composition of the meal are relevant and could be potentially manipulated for therapeutic purposes. Modern digital tools can provide monitoring capability of feeding and fasting habits over the 24-h period and a way to behavioural dietary interventions (16, 86, 97–99).

Furthermore, diet can influence markers of immune function, with an association between diet and incidence of several immune-mediated diseases including allergy, diabetes, and cancer reported (100). Moreover, fasting can influence immune responses in tumour-laden mice, with twice-monthly fasting resulting in higher white blood cell count and reduction in neoplasms despite no change in calorie intake (101). The influence of circadian dietary pattern on the immune system has also been explored, with studies showing associations between circadian feeding cycle, fasting period and alterations in both adaptive and innate immune response, with potential therapeutic implications (56, 102, 103).

Modifying diet also affects the gut microbiome, with different diets associated with noticeably different abundances and diversity of gut microbiota (104–106).

Interactions between outcomes on ICI and diet are thought to often occur via the microbiome, with studies reporting correlations between diversity and relative abundance of specific species, such as *Akkermansia* and *Ruminococcaceae* (107–109).

Contrarily to light exposure and physical exercise, there is clinical evidence on the impact of diet type on ICI efficacy. Although there is some discordance between studies, and heterogeneity with regards to cut-offs, adherence and duration of particular diets, disease types and clinical outcomes, there is an overall trend towards better outcomes associated with what is regarded as an healthy diet in humans in general by the WHO (110). For instance, high amount of fruit and vegetable, and low amount of dairy portions, were significantly associated with clinical benefit from ICI therapy (111). Specifically, increased fibre intake (threshold of 20g per day or more), higher adherence to a Mediterranean diet (rich in whole grains, fish, nuts, fruit, and vegetables, and low in red and processed meat), and a periodic fasting-mimicking diet (consisting of a nutritional composition that mimics fasting) displayed beneficial impact in patients receiving ICI in various studies (112–114).

Moreover, normal (> 30 ng/dL) vitamin D3 levels, whether naturally-occurring or through oral supplementation, were associated with significantly better outcomes (115). Furthermore, experimental evidence suggests an impact of ketogenic (low carbohydrate, low protein, and high fat) diet, of dietary amino-acid restriction and of polyphenols administration on ICI efficacy (116–118).

Interestingly, defecation frequency was also relevant, with emptying bowels less than daily associated with poor response to ICI (111).

## Discussion

Immune checkpoint inhibitors have provided enormous benefit in the management of an ever-expanding array of cancer types, with dramatic increases in overall survival in those who respond. Their current main weaknesses lie in a variable response rate and risk of toxicity. Recent studies have consistently reported increased efficacy of ICI therapy when infusions were administered in the morning, and that timing of immunotherapy is an independent prognostic factor for overall survival (10, 51, 119–126). This suggests a link between ICI efficacy and the CTS, which is responsible for circadian variations in many physiological features. In reporting a correlation between time of administration and efficacy, the findings suggest the CTS could be harnessed by clinicians to improve ICI efficacy. In order to do this, it is important a patient’s CTS is entrained, as any benefit could be impaired by CTS disruption. Indeed, circadian disruption (evaluated with continuous wrist-actigraphy or with diurnal salivary cortisol slope) has been associated with shorter overall survival in various cancers, but not yet in those treated with ICI (127, 128).

Conveniently, the CTS can be entrained by numerous lifestyle factors which have also been shown in studies to have independent effects on the immune system, the microbiome and sometimes on ICI efficacy, as shown above and as brilliantly discussed by others very recently (13). Both ICI therapy and circadian systems are complex, and further research will be needed to better understand the science of their interactions in order to harness this insight for therapeutic purposes.

Although extensive research is aiming at identifying tumour-associated or host-related factors predicting for ICI efficacy or tolerability, most of them are immutable and intrinsic to the patient and the disease, thus potentially impossible to be manipulated (e.g., PD-L1 expression levels) or very hard to be meaningfully modified in a relatively short timeframe (e.g., body mass index) (129). Similarly, the use of some drugs (e.g., antibiotics, proton-pump inhibitors and obviously steroids) has been shown to impair ICI efficacy in retrospective studies (130). Yet, most likely these drugs have been prescribed for a therapeutic reason and arguably it would not be easy to avoid them altogether in clinical practice.

Conversely, lifestyle interventions, including light exposure, physical exercise and diet, could be allegedly manipulated more easily to obtain the maximal therapeutic benefit from ICI (**Table 1**). Thus, a circadian-based optimisation of entraining cues and timing of administration could safely improve the outcomes of cancer patients treated with ICI.

**Table 1:** Table of potential lifestyle interventions, their effects, and clinical considerations for studies/deployment.

Intervention	Effects	Aspects to be considered/optimised
**Physical Exercise**	* Entrainment of the CTS, increased production of melatonin and lower cortisol release at night (82) * Anti-inflammatory and immunoregulatory effects (91) * Immune cell mobilisation to peripheral bloodstream, including PD-1 CD8+ T cells, which are vital for host tumour defence (88, 89) * Enrichment of gut microbiome (91)	* Regularity and frequency of exercise * Time of day exercise is conducted * Intensity and type of exercise * Duration of exercise * Circadian phase whilst exercising and change in body temperature
**Light Exposure**	* Causes suprachiasmatic nucleus neurons (master clock) to alter clock gene expression (53, 131, 132) * Clock genes expressed synchronise peripheral clocks to the daily light dark cycle (120, 133) * Affects both innate and adaptive immunity (43, 45, 57, 134) * Alters gut microbiome and its metabolic activity (69, 135)	* Timings of bright (outdoors) light exposure * Duration of bright (outdoors) light exposure * Intensity of artificial bright light exposure * Timing of avoidance of artificial light at night exposure * Feasible and realistic intensity (and spectrum) of acceptable artificial light at night
**Diet**	* Entrainment of the CTS (94) * High levels of fruit and veg consumption associated with improved ICI efficacy (111) * Low dairy consumption also associated with improved ICI efficacy (111) * Increased fibre intake associated with improved progression-free survival (112) * Normal vitamin D levels (with or without supplementation) associated with improved response rate (115) * Increased adherence to Mediterranean diet associated with increased chance of response to ICI (114) * Fasting-mimicking diet associated with increased ICI efficacy (113) * Opening bowels daily associated with improved ICI efficacy (111)	* Fibre intake * Vitamin D levels * Mediterranean diet adherence * Fruit and vegetable consumption * Dairy consumption * Frequency of defecation * Spacing of meals throughout day * Duration of fasting period * Consistency of meal timings

However, this would require dedicated observational and interventional studies, with a robust translational component, in order to precisely and dynamically personalise lifestyle modifications. Indeed, the intertwining between these factors are multiple and complex, and involve hormonal messaging (e.g., melatonin, Vitamin D), unavoidable interactions (e.g., between outdoors physical activity and exposure to bright sunlight), and indirect microbiome-mediated mechanisms.

Further, they are all intrinsically bound to occur at a certain time of the day, thus impacting on the CTS and its temporal control of the immune system and of pharmacological determinants (136, 137).

Thus, although with this brief overview we have critically discussed photic stimuli, physical exercise and dietary factors, encompassing clinical and experimental findings, we believe that additional research will be of great interest and should be warranted in furthering our understanding of the effects of lifestyle factors on ICI efficacy as a whole, through modulation of the CTS, and the temporal organisation of the immune system and the microbiota.

However, difficulties with this approach should be acknowledged, including the intrinsic heterogeneity in populations, studies and outcomes, and, for instance, microbiome composition across cohorts (138), as well as the tolerance to interventions to factor into a patient’s cancer treatment plan.

This tolerance is indeed equally relevant when using lifestyle modifications as treatments. Consideration should be given as to the likeliness of patients with cancer and undergoing cancer treatment being able to enact and maintain lifestyle changes without unduly impacting their quality of life. For the patient, the impact of certain lifestyle modifications may not outweigh the possible benefit of increased ICI efficacy in a trade-off which will be personal to the patient.

It is difficult to discuss the potential for lifestyle modifications to optimise cancer treatment further without taking the time to emphasise the importance of the individual patient. Not only will optimisation of cancer treatment have to consider cancer subtype and patient chronotype, but also the patient’s symptoms, comorbidities, habits, health beliefs, socio-economic status, social support, self-determination, and values in helping them make informed decisions on how best to utilise lifestyle modifications to optimise their cancer management. Practical implementation of such approaches could also be challenging, without appropriate support. Tellingly, surveys carried out exploring how often patients implement lifestyle changes after cancer diagnosis found that 41 to 65% of patients made dietary changes post-cancer diagnosis and 14 to 27% increased their level of exercise (139, 140). Future research could also make use of digital technologies to monitor circadian biorhythm to further refine our understanding of the correlation between the CTS and outcomes on ICI (127).

In summary, building on evidence showing the CTS plays a role in increasing ICI efficacy and circadian disruption have deleterious effect on cancer patients survival, we argue CTS precise and personalised entrainment by lifestyle factors such as photic stimuli, diet composition and timing, and physical exercise could be harnessed to potentially increase ICI efficacy. Conveniently, existing evidence suggests these behavioural interventions shown to improve outcomes on ICI – either directly or via the gut microbiota – regularly are associated with healthier lifestyle habits, with intrinsic health benefits. Combining these findings, the CTS could feasibly be entrained by a patient-tailored combination of lifestyle determinants of ICI efficacy to maximise response, with future research offering patients and clinicians an expanding evidence base on which to draw from.

## Author contributions

BH: Data curation, Formal Analysis, Investigation, Methodology, Resources, Visualization, Writing – original draft, Writing – review & editing. SS: Data curation, Formal Analysis, Investigation, Resources, Writing – original draft, Writing – review & editing. SI: Data curation, Formal Analysis, Investigation, Resources, Writing – original draft, Writing – review & editing. NW: Conceptualization, Data curation, Formal Analysis, Funding acquisition, Investigation, Methodology, Resources, Supervision, Validation, Visualization, Writing – original draft, Writing – review & editing. CS: Formal Analysis, Funding acquisition, Methodology, Supervision, Validation, Writing – review & editing. PI: Conceptualization, Data curation, Formal Analysis, Funding acquisition, Investigation, Methodology, Project administration, Resources, Supervision, Validation, Visualization, Writing – original draft, Writing – review & editing.

## References

[1] Ledford H Else H Warren M. Cancer immunologists scoop medicine Nobel prize. Nature (2018) 562(7725):20–1. doi: 10.1038/d41586-018-06751-0 30279600

[2] Halliday GM Patel A Hunt MJ Tefany FJ Barnetson RSC. Spontaneous regression of human melanoma/nonmelanoma skin cancer: Association with infiltrating CD4+ T cells. World J Surg (1995) 19(3):352–8. doi: 10.1007/BF00299157 7638987

[3] Seidel JA Otsuka A Kabashima K. Anti-PD-1 and anti-CTLA-4 therapies in cancer: mechanisms of action, efficacy, and limitations. Front Oncol (2018) 8:86. doi: 10.3389/fonc.2018.00086 29644214 PMC5883082

[4] Korman AJ Garrett-Thomson SC Lonberg N. The foundations of immune checkpoint blockade and the ipilimumab approval decennial. Nat Rev Drug Discovery (2022) 21(7):509–28. doi: 10.1038/s41573-021-00345-8 34937915

[5] Morad G Helmink BA Sharma P Wargo JA. Hallmarks of response, resistance, and toxicity to immune checkpoint blockade. Cell (2021) 184(21):5309–37. doi: 10.1016/j.cell.2021.09.020 PMC8767569 34624224

[6] Postow MA Sidlow R Hellmann MD. Immune-related adverse events associated with immune checkpoint blockade. N Engl J Med (2018) 378(2):158–68. doi: 10.1056/NEJMra1703481 29320654

[7] Johnson DB Nebhan CA Moslehi JJ Balko JM. Immune-checkpoint inhibitors: long-term implications of toxicity. Nat Rev Clin Oncol (2022) 19(4):254–67. doi: 10.1038/s41571-022-00600-w PMC8790946 35082367

[8] Gibney GT Weiner LM Atkins MB. Predictive biomarkers for checkpoint inhibitor-based immunotherapy. Lancet Oncol (2016) 17(12):e542–51. doi: 10.1016/S1470-2045(16)30406-5 PMC5702534 27924752

[9] Meyers DE Banerji S. Biomarkers of immune checkpoint inhibitor efficacy in cancer. Curr Oncol (2020) 27:106–14. doi: 10.3747/co.27.5549 PMC7194000 32368180

[10] Karaboué A Collon T Pavese I Bodiguel V Cucherousset J Zakine E. Time-dependent efficacy of checkpoint inhibitor nivolumab: results from a pilot study in patients with metastatic non-small-cell lung cancer. Cancers (2022) 14:896. doi: 10.3390/cancers14040896 35205644 PMC8870559

[11] Golombek DA Rosenstein RE. Physiology of circadian entrainment. Physiol Rev (2010) 90(3):1063–102. doi: 10.1152/physrev.00009.2009 20664079

[12] Elvira A-L. The circadian timing system: A recent addition in the physiological mechanisms underlying pathological and aging processes. Aging Dis (2014) 5(6):406–18. doi: 10.14336/AD.2014.0500406 PMC4249810 25489492

[13] Boesch M Baty F Rassouli F Kowatsch T Joerger M Früh M. Non-pharmaceutical interventions to optimize cancer immunotherapy. Oncoimmunology (2023) 12(1):2255459. doi: 10.1080/2162402X.2023.2255459 37791231 PMC10543347

[14] Clark E Maguire H Cannon P Leung EY. The effects of physical activity, fast-mimicking diet and psychological interventions on cancer survival: A systematic review and meta-analysis of randomized controlled trials. Complement Ther Med (2021) 57:102654. doi: 10.1016/j.ctim.2020.102654 33359756 PMC8047871

[15] McCorkle R Ercolano E Lazenby M Schulman-Green D Schilling LS Lorig K. Self-management: Enabling and empowering patients living with cancer as a chronic illness. CA Cancer J Clin (2011) 61(1):50–62. doi: 10.3322/caac.20093 21205833 PMC3058905

[16] Gibb M Winter H Komarzynski S Wreglesworth NI Innominato PF. Holistic needs assessment of cancer survivors—Supporting the process through digital monitoring of circadian physiology. Integr Cancer Ther (2022) 21:15347354221123524. doi: 10.1177/15347354221123525 PMC9520145 36154506

[17] Allada R Bass J. Circadian mechanisms in medicine. N Engl J Med (2021) 384(6):550–61. doi: 10.1056/NEJMra1802337 PMC8108270 33567194

[18] Roenneberg T Merrow M. The circadian clock and human health. Curr Biol (2016) 26(10):R432–43. doi: 10.1016/j.cub.2016.04.011 27218855

[19] Patke A Young MW Axelrod S. Molecular mechanisms and physiological importance of circadian rhythms. Nat Rev Mol Cell Biol (2020) 21(2):67–84. doi: 10.1038/s41580-019-0179-2 31768006

[20] Ukai H Ueda HR. Systems biology of mammalian circadian clocks. Annu Rev Physiol (2010) 72:579–603. doi: 10.1146/annurev-physiol-073109-130051 20148689

[21] Ortega-Campos SM Verdugo-Sivianes EM Amiama-Roig A Blanco JR Carnero A. Interactions of circadian clock genes with the hallmarks of cancer. Biochim Biophys Acta Rev Cancer (2023) 1878(3):188900. doi: 10.1016/j.bbcan.2023.188900 37105413

[22] Mirian M Hariri A Yadollahi M Kohandel M. Circadian and immunity cycle talk in cancer destination: from biological aspects to in silico analysis. Cancers (Basel) (2022) 14(6):1578. doi: 10.3390/cancers14061578 35326729 PMC8945968

[23] Frank E Wallace ML Matthews MJ Kendrick J Leach J Moore T. Personalized digital intervention for depression based on social rhythm principles adds significantly to outpatient treatment. Front Digit Heal (2022) 4:870522. doi: 10.3389/fdgth.2022.870522 PMC9478192 36120713

[24] Roenneberg T Merrow M. Entrainment of the human circadian clock. Cold Spring Harb Symp Quant Biol (2007) 72:293–9. doi: 10.1101/sqb.2007.72.043 18419286

[25] Wehrens SMT Christou S Isherwood C Middleton B Gibbs MA Archer SN. Meal timing regulates the human circadian system. Curr Biol (2017) 27(12):1768–1775.e3. doi: 10.1016/j.cub.2017.04.059 28578930 PMC5483233

[26] Huang Y Mayer C Walch OJ Bowman C Sen S Goldstein C. Distinct circadian assessments from wearable data reveal social distancing promoted internal desynchrony between circadian markers. Front Digit Heal (2021) 3:727504. doi: 10.3389/fdgth.2021.727504 PMC8634937 34870267

[27] Jamshed H Beyl RA Della Manna DL Yang ES Ravussin E Peterson CM. Early time-restricted feeding improves 24-hour glucose levels and affects markers of the circadian clock, aging, and autophagy in humans. Nutrients (2019) 11:1234. doi: 10.3390/nu11061234 31151228 PMC6627766

[28] Amidi A Wu LM. Circadian disruption and cancer- and treatment-related symptoms. Front Oncol (2022) 12:1009064. doi: 10.3389/fonc.2022.1009064 36387255 PMC9650229

[29] Scheiermann C Gibbs J Ince L Loudon A. Clocking in to immunity. Nat Rev Immunol (2018) 18(7):423–37. doi: 10.1038/s41577-018-0008-4 29662121

[30] Logan RW Zhang C Murugan S O’Connell S Levitt D Rosenwasser AM. Chronic shift-lag alters the circadian clock of NK cells and promotes lung cancer growth in rats. J Immunol (2012) 188(6):2583–91. doi: 10.4049/jimmunol.1102715 PMC3294088 22308312

[31] Aiello I Fedele MLM Román F Marpegan L Caldart C Chiesa JJ. Circadian disruption promotes tumor-immune microenvironment remodeling favoring tumor cell proliferation. Sci Adv (2020) 6(42):eaaz4530. doi: 10.1126/sciadv.aaz4530 33055171 PMC7556830

[32] Hadadi E Taylor W Li X-M Aslan Y Villote M Rivière J. Chronic circadian disruption modulates breast cancer stemness and immune microenvironment to drive metastasis in mice. Nat Commun (2020) 11(1):3193. doi: 10.1038/s41467-020-16890-6 32581213 PMC7314789

[33] Wang C Barnoud C Cenerenti M Sun M Caffa I Kizil B. Dendritic cells direct circadian anti-tumour immune responses. Nature (2023) 614(7946):136–43. doi: 10.1038/s41586-022-05605-0 PMC9891997 36470303

[34] Faraut B Cordina-Duverger E Aristizabal G Drogou C Gauriau C Sauvet F. Immune disruptions and night shift work in hospital healthcare professionals: The intricate effects of social jet-lag and sleep debt. Front Immunol (2022) 13:939829. doi: 10.3389/fimmu.2022.939829 36164341 PMC9509137

[35] Lévi F A Okyar A Hadadi E F Innominato P Ballesta A. Circadian regulation of drug responses: toward sex-specific and personalized chronotherapy. Annu Rev Pharmacol Toxicol (2023) 64. doi: 10.1146/annurev-pharmtox-051920-095416 37722720

[36] Haspel JA Anafi R Brown MK Cermakian N Depner C Desplats P. Perfect timing: circadian rhythms, sleep, and immunity - an NIH workshop summary. JCI Insight (2020) 5(1):e131487. doi: 10.1172/jci.insight.131487 31941836 PMC7030790

[37] Thomas AM Fidelle M Routy B Kroemer G Wargo JA Segata N. Gut OncoMicrobiome Signatures (GOMS) as next-generation biomarkers for cancer immunotherapy. Nat Rev Clin Oncol (2023) 20(9):583–603. doi: 10.1038/s41571-023-00785-8 37365438 PMC11258874

[38] Litichevskiy L Thaiss CA. The oscillating gut microbiome and its effects on host circadian biology. Annu Rev Nutr (2022) 42:145–64. doi: 10.1146/annurev-nutr-062320-111321 35576592

[39] Fishbein AB Knutson KL Zee PC. Circadian disruption and human health. J Clin Invest (2021) 131(19):e148286. doi: 10.1172/JCI148286 34596053 PMC8483747

[40] Durack J Lynch SV. The gut microbiome: Relationships with disease and opportunities for therapy. J Exp Med (2019) 216(1):20–40. doi: 10.1084/jem.20180448 30322864 PMC6314516

[41] Guillot N Roméo B Manesh SS Milano G Brest P Zitvogel L. Manipulating the gut and tumor microbiota for immune checkpoint inhibitor therapy: from dream to reality. Trends Mol Med (2023) 29(11):897–911. doi: 10.1016/j.molmed.2023.08.004 37704493

[42] Gutierrez Lopez DE Lashinger LM Weinstock GM Bray MS. Circadian rhythms and the gut microbiome synchronize the host's metabolic response to diet. Cell Metab (2021) 33(5):873–87. doi: 10.1016/j.cmet.2021.03.015 33789092

[43] Walker WH 2nd Bumgarner JR Becker-Krail DD May LE Liu JA Nelson RJ. Light at night disrupts biological clocks, calendars, and immune function. Semin Immunopathol (2022) 44(2):165–73. doi: 10.1007/s00281-021-00899-0 PMC8564795 34731290

[44] Blume C Garbazza C Spitschan M. Effects of light on human circadian rhythms, sleep and mood. Somnol Schlafforsch Und Schlafmedizin Somnol Sleep Res Sleep Med (2019) 23(3):147–56. doi: 10.1007/s11818-019-00215-x PMC6751071 31534436

[45] Panshikar S Haldar C. Immune responses of Indian Jungle Bush Quail, P. asiatica, to different photoperiodic regimens during the reproductively inactive phase. Biol Rhythm Res (2009) 40(3):235–47. doi: 10.1080/09291010701875328

[46] Simpson RC Shanahan ER Scolyer RA Long GV. Towards modulating the gut microbiota to enhance the efficacy of immune-checkpoint inhibitors. Nat Rev Clin Oncol (2023) 20(10):697–715. doi: 10.1038/s41571-023-00803-9 37488231

[47] Masri S Sassone-Corsi P. The emerging link between cancer, metabolism, and circadian rhythms. Nat Med (2018) 24(12):1795–803. doi: 10.1038/s41591-018-0271-8 PMC6535395 30523327

[48] Ma Y Kroemer G. The cancer-immune dialogue in the context of stress. Nat Rev Immunol (2023). doi: 10.1038/s41577-023-00949-8 37833492

[49] Bishehsari F Voigt RM Keshavarzian A. Circadian rhythms and the gut microbiota: from the metabolic syndrome to cancer. Nat Rev Endocrinol (2020) 16(12):731–9. doi: 10.1038/s41574-020-00427-4 PMC8085809 33106657

[50] Li X Zhang S Guo G Han J Yu J. Gut microbiome in modulating immune checkpoint inhibitors. eBioMedicine (2022) 82:104163. doi: 10.1016/j.ebiom.2022.104163 35841869 PMC9297075

[51] Landre T Karaboue A Buchwald ZS Innominato P Qian DC Assie J. Time-dependent efficacy of immune checkpoint inhibitors in the treatment of metastatic cancers: A meta-analysis. J Clin Oncol (2023) 41(16_suppl):2562. doi: 10.1200/JCO.2023.41.16_suppl.2562

[52] Walker WH Borniger JC Gaudier-Diaz MM Hecmarie Meléndez-Fernández O Pascoe JL Courtney DeVries A. Acute exposure to low-level light at night is sufficient to induce neurological changes and depressive-like behavior. Mol Psychiatry (2020) 25(5):1080–93. doi: 10.1038/s41380-019-0430-4 PMC6881534 31138889

[53] Wood S Loudon A. Clocks for all seasons: unwinding the roles and mechanisms of circadian and interval timers in the hypothalamus and pituitary. J Endocrinol (2014) 222(2):R39–59. doi: 10.1530/JOE-14-0141 PMC4104039 24891434

[54] Leach S Suzuki K. Adrenergic signaling in circadian control of immunity. Front Immunol (2020) 11:1235. doi: 10.3389/fimmu.2020.01235 32714319 PMC7344327

[55] Dimitrov S Benedict C Heutling D Westermann J Born J Lange T. Cortisol and epinephrine control opposing circadian rhythms in T cell subsets. Blood (2009) 113(21):5134–43. doi: 10.1182/blood-2008-11-190769 PMC2686184 19293427

[56] Blaževitš O Di Tano M Longo VD. Fasting and fasting mimicking diets in cancer prevention and therapy. Trends Cancer (2023) 9(3):212–22. doi: 10.1016/j.trecan.2022.12.006 36646607

[57] Okuliarova M Mazgutova N Majzunova M Rumanova VS Zeman M. Dim light at night impairs daily variation of circulating immune cells and renal immune homeostasis. Front Immunol (2021) 11:614960. doi: 10.3389/fimmu.2020.614960 33552079 PMC7862740

[58] Dollish HK Tsyglakova M McClung CA. Circadian rhythms and mood disorders: Time to see the light. Neuron (2023). doi: 10.1016/j.neuron.2023.09.023 PMC10842077 37858331

[59] Yoshiike T Dallaspezia S Kuriyama K Yamada N Colombo C Benedetti F. Association of circadian properties of temporal processing with rapid antidepressant response to wake and light therapy in bipolar disorder. J Affect Disord (2020) 263:72–9. doi: 10.1016/j.jad.2019.11.132 31818799

[60] Meesters Y van Tuinen EJD Gordijn MCM. 35 years of light treatment for mental disorders in the Netherlands. Ann Med (2023) 55(2):2269574. doi: 10.1080/07853890.2023.2269574 37857364 PMC10588530

[61] Mayer C Walch O Forger DB Hannay K. Impact of light schedules and model parameters on the circadian outcomes of individuals. J Biol Rhythms (2023) 38(4):379–91. doi: 10.1177/07487304231176936 37350312

[62] Huang Y Mayer C Cheng P Siddula A Burgess HJ Drake C. Predicting circadian phase across populations: a comparison of mathematical models and wearable devices. Sleep (2021) 44(10):zsab126. doi: 10.1093/sleep/zsab126 34013347 PMC8503830

[63] Pick R He W Chen C-S Scheiermann C. Time-of-day-dependent trafficking and function of leukocyte subsets. Trends Immunol (2019) 40(6):524–37. doi: 10.1016/j.it.2019.03.010 31109762

[64] Druzd D Matveeva O Ince L Harrison U He W Schmal C. Lymphocyte circadian clocks control lymph node trafficking and adaptive immune responses. Immunity (2017) 46(1):120–32. doi: 10.1016/j.immuni.2016.12.011 PMC5263259 28087238

[65] Chiu K Warner G Nowak RA Flaws JA Mei W. The impact of environmental chemicals on the gut microbiome. Toxicol Sci (2020) 176(2):253–84. doi: 10.1093/toxsci/kfaa065 PMC7416318 32392306

[66] Malinowska K Szala K Podkowa P Surmacki A. Effect of light intensity in the nest site on eggshell pigmentation in a hole-nesting passerine. Sci Rep (2023) 13(1):9764. doi: 10.1038/s41598-023-36658-4 37328505 PMC10276044

[67] González Maglio DH Paz ML Leoni J. Sunlight effects on immune system: is there something else in addition to UV-induced immunosuppression? BioMed Res Int (2016) 2016:1934518. doi: 10.1155/2016/1934518 28070504 PMC5187459

[68] Petrowski K Schmalbach B Linhardt M Mekschrat L Rohleder N. The inflammatory immune system after wake up in healthy male individuals: A highly standardized and controlled study. Brain Behav Immun Heal (2022) 25:100504. doi: 10.1016/j.bbih.2022.100504 PMC9450065 36093437

[69] Wu G Tang W He Y Hu J Gong S He Z. Light exposure influences the diurnal oscillation of gut microbiota in mice. Biochem Biophys Res Commun (2018) 501(1):16–23. doi: 10.1016/j.bbrc.2018.04.095 29730287

[70] Carrillo-Vico A Lardone PJ Alvarez-Sánchez N Rodríguez-Rodríguez A Guerrero JM. Melatonin: buffering the immune system. Int J Mol Sci (2013) 14(4):8638–83. doi: 10.3390/ijms14048638 PMC3645767 23609496

[71] Won E Na K-S Kim Y-K. Associations between melatonin, neuroinflammation, and brain alterations in depression. Int J Mol Sci (2021) 23(1):305. doi: 10.3390/ijms23010305 35008730 PMC8745430

[72] Bikle DD. Vitamin D and the skin: Physiology and pathophysiology. Rev Endocr Metab Disord (2012) 13(1):3–19. doi: 10.1007/s11154-011-9194-0 21845365 PMC3687803

[73] Segaert S Simonart T. The epidermal vitamin D system and innate immunity: some more light shed on this unique photoendocrine system? Dermatol (Basel Switzerland) (2008) 217:7–11. doi: 10.1159/000118506 18309238

[74] Singh MM Mullin GE. 115Diet, environmental chemicals, and the gut microbiome. In: Cohen A vom Saal FS Weil A, editors. Integrative Environmental Medicine. New York: Oxford University Press (2017). doi: 10.1093/med/9780190490911.003.0006

[75] Fletcher J Cooper SC Ghosh S Hewison M. The role of vitamin D in inflammatory bowel disease: mechanism to management. Nutrients (2019) 11(5):1019. doi: 10.3390/nu11051019 31067701 PMC6566188

[76] Bennour I Haroun N Sicard F Mounien L Landrier J-F. Vitamin D and obesity/adiposity-A brief overview of recent studies. Nutrients (2022) 14(10):2049. doi: 10.3390/nu14102049 35631190 PMC9143180

[77] Lips P Eekhoff M van Schoor N Oosterwerff M de Jongh R Krul-Poel Y. Vitamin D and type 2 diabetes. J Steroid Biochem Mol Biol (2017) 173:280–5. doi: 10.1016/j.jsbmb.2016.11.021 27932304

[78] Roffe-Vazquez DN Huerta-Delgado AS Castillo EC Villarreal-Calderón JR Gonzalez-Gil AM Enriquez C. Correlation of vitamin D with inflammatory cytokines, atherosclerotic parameters, and lifestyle factors in the setting of heart failure: A 12-month follow-up study. Int J Mol Sci (2019) 20(22):5811. doi: 10.3390/ijms20225811 31752330 PMC6887713

[79] Sun J Zhang Y-G. Vitamin D receptor influences intestinal barriers in health and disease. Cells (2022) 11(7):1129. doi: 10.3390/cells11071129 35406694 PMC8997406

[80] Singh P Rawat A Alwakeel M Sharif E Al Khodor S. The potential role of vitamin D supplementation as a gut microbiota modifier in healthy individuals. Sci Rep (2020) 10(1):21641. doi: 10.1038/s41598-020-77806-4 33303854 PMC7729960

[81] Sîrbe C Rednic S Grama A Pop TL. An update on the effects of vitamin D on the immune system and autoimmune diseases. Int J Mol Sci (2022) 23(17):9784. doi: 10.3390/ijms23179784 36077185 PMC9456003

[82] Hower IM Harper SA Buford TW. Circadian rhythms, exercise, and cardiovascular health. J Circadian Rhythms (2018) 16:7. doi: 10.5334/jcr.164 30210567 PMC6083774

[83] Shen B Ma C Wu G Liu H Chen L Yang G. Effects of exercise on circadian rhythms in humans. Front Pharmacol (2023) 14:1282357. doi: 10.3389/fphar.2023.1282357 37886134 PMC10598774

[84] Silva BS de A Uzeloto JS Lira FS Pereira T Coelho-E-Silva MJ Caseiro A. Exercise as a peripheral circadian clock resynchronizer in vascular and skeletal muscle aging. Int J Environ Res Public Health (2021) 18(24):12949. doi: 10.3390/ijerph182412949 34948558 PMC8702158

[85] Yamanaka Y Honma K Hashimoto S Takasu N Miyazaki T Honma S. Effects of physical exercise on human circadian rhythms. Sleep Biol Rhythms (2006) 4(3):199–206. doi: 10.1111/j.1479-8425.2006.00234.x

[86] Bowman C Huang Y Walch OJ Fang Y Frank E Tyler J. A method for characterizing daily physiology from widely used wearables. Cell Rep Methods (2021) 1(4):100058. doi: 10.1016/j.crmeth.2021.100058 34568865 PMC8462795

[87] Ester M Eisele M Wurz A McDonough MH McNeely M Culos-Reed SN. Current evidence and directions for future research in eHealth physical activity interventions for adults affected by cancer: systematic review. JMIR Cancer (2021) 7(3):e28852. doi: 10.2196/28852 34542415 PMC8491123

[88] Gouez M Pérol O Pérol M Caux C Ménétrier-Caux C Villard M. Effect of acute aerobic exercise before immunotherapy and chemotherapy infusion in patients with metastatic non-small-cell lung cancer: protocol for the ERICA feasibility trial. BMJ Open (2022) 12(4):e056819. doi: 10.1136/bmjopen-2021-056819 PMC8990709 35393316

[89] Lasvergnas J Fallet V Duchemann B Jouveshomme S Cadranel J Chouaïd C. PDL1-status predicts primary resistance of metastatic, EGFR-mutated non small cell lung cancers to EGFR tyrosine-kinase inhibitors. Respir Med Res (2023) 84:101018. doi: 10.1016/j.resmer.2023.101018 37302160

[90] Kurz E Hirsch CA Dalton T Shadaloey SA Khodadadi-Jamayran A Miller G. Exercise-induced engagement of the IL-15/IL-15Rα axis promotes anti-tumor immunity in pancreatic cancer. Cancer Cell (2022) 40(7):720–737.e5. doi: 10.1016/j.ccell.2022.05.006 35660135 PMC9280705

[91] Dziewiecka H Buttar HS Kasperska A Ostapiuk–Karolczuk J Domagalska M Cichoń J. Physical activity induced alterations of gut microbiota in humans: a systematic review. BMC Sports Sci Med Rehabil (2022) 14(1):122. doi: 10.1186/s13102-022-00513-2 35799284 PMC9264679

[92] Vivarelli S Salemi R Candido S Falzone L Santagati M Stefani S. Gut microbiota and cancer: from pathogenesis to therapy. Cancers (Basel) (2019) 11(1):38. doi: 10.3390/cancers11010038 30609850 PMC6356461

[93] Sato T Sassone-Corsi P. Nutrition, metabolism, and epigenetics: pathways of circadian reprogramming. EMBO Rep (2022) 23(5):e52412. doi: 10.15252/embr.202152412 35412705 PMC9066069

[94] Tahara Y Shibata S. Chronobiology and nutrition. Neuroscience (2013) 253:78–88. doi: 10.1016/j.neuroscience.2013.08.049 24007937

[95] Eckel-Mahan KL Patel VR de Mateo S Orozco-Solis R Ceglia NJ Sahar S. Reprogramming of the circadian clock by nutritional challenge. Cell (2013) 155(7):1464–78. doi: 10.1016/j.cell.2013.11.034 PMC4573395 24360271

[96] Itokawa M Hirao A Nagahama H Otsuka M Ohtsu T Furutani N. Time-restricted feeding of rapidly digested starches causes stronger entrainment of the liver clock in PER2::LUCIFERASE knock-in mice. Nutr Res (2013) 33(2):109–19. doi: 10.1016/j.nutres.2012.12.004 23399661

[97] Christensen S Huang Y Walch OJ Forger DB. Optimal adjustment of the human circadian clock in the real world. PloS Comput Biol (2020) 16(12):e1008445. doi: 10.1371/journal.pcbi.1008445 33370265 PMC7808694

[98] Bae S-A Fang MZ Rustgi V Zarbl H Androulakis IP. At the interface of lifestyle, behavior, and circadian rhythms: metabolic implications. Front Nutr (2019) 6:132. doi: 10.3389/fnut.2019.00132 31555652 PMC6722208

[99] Wang L Langlais CS Kenfield SA Chan JM Graff RE Allen IE. mHealth interventions to promote a healthy diet and physical activity among cancer survivors: A systematic review of randomized controlled trials. Cancers (Basel) (2022) 14(15):3816. doi: 10.3390/cancers14153816 35954479 PMC9367623

[100] Venter C Eyerich S Sarin T Klatt KC. Nutrition and the immune system: A complicated tango. Nutrients (2020) 12(3):818. doi: 10.3390/nu12030818 32204518 PMC7146186

[101] Longo VD Panda S. Fasting, circadian rhythms, and time-restricted feeding in healthy lifespan. Cell Metab (2016) 23(6):1048–59. doi: 10.1016/j.cmet.2016.06.001 PMC5388543 27304506

[102] Zheng D Ratiner K Elinav E. Circadian influences of diet on the microbiome and immunity. Trends Immunol (2020) 41(6):512–30. doi: 10.1016/j.it.2020.04.005 32359722

[103] Cortellino S Quagliariello V Delfanti G Blaževitš O Chiodoni C Maurea N. Fasting mimicking diet in mice delays cancer growth and reduces immunotherapy-associated cardiovascular and systemic side effects. Nat Commun (2023) 14(1):5529. doi: 10.1038/s41467-023-41066-3 37684243 PMC10491752

[104] Bibbò S Ianiro G Giorgio V Scaldaferri F Masucci L Gasbarrini A. The role of diet on gut microbiota composition. Eur Rev Med Pharmacol Sci (2016) 20(22):4742–9. 27906427

[105] Beam A Clinger E Hao L. Effect of diet and dietary components on the composition of the gut microbiota. Nutrients (2021) 13(8):2795. doi: 10.3390/nu13082795 34444955 PMC8398149

[106] Wang X Geng S. Diet-gut microbial interactions influence cancer immunotherapy. Front Oncol (2023) 13:1138362. doi: 10.3389/fonc.2023.1138362 37035188 PMC10081683

[107] Jin Y Dong H Xia L Yang Y Zhu Y Shen Y. The diversity of gut microbiome is associated with favorable responses to anti–Programmed death 1 immunotherapy in chinese patients with NSCLC. J Thorac Oncol (2019) 14(8):1378–89. doi: 10.1016/j.jtho.2019.04.007 31026576

[108] Zheng Y Wang T Tu X Huang Y Zhang H Tan D. Gut microbiome affects the response to anti-PD-1 immunotherapy in patients with hepatocellular carcinoma. J Immunother Cancer (2019) 7(1):193. doi: 10.1186/s40425-019-0650-9 31337439 PMC6651993

[109] Derosa L Routy B Thomas AM Iebba V Zalcman G Friard S. Intestinal Akkermansia muciniphila predicts clinical response to PD-1 blockade in patients with advanced non-small-cell lung cancer. Nat Med (2022) 28(2):315–24. doi: 10.1038/s41591-021-01655-5 PMC9330544 35115705

[110] World Health Organisation. WHO fact sheet. In: Healthy diet (Geneva, Switzerland: World Health Organization) (2018). Available at: https://www.who.int/en/news-room/fact-sheets/detail/healthy-diet.

[111] Pietrzak B Tomela K Olejnik-Schmidt A Galus Ł Mackiewicz J Kaczmarek M. A clinical outcome of the anti-PD-1 therapy of melanoma in polish patients is mediated by population-specific gut microbiome composition. Cancers (2022) 14(21):5369. doi: 10.3390/cancers14215369 36358789 PMC9653730

[112] Spencer CN McQuade JL Gopalakrishnan V McCulloch JA Vetizou M Cogdill AP. Dietary fiber and probiotics influence the gut microbiome and melanoma immunotherapy response. Sci (80 ) (2021) 374(6575):1632–40. doi: 10.1126/science.aaz7015 PMC8970537 34941392

[113] Cortellino S Raveane A Chiodoni C Delfanti G Pisati F Spagnolo V. Fasting renders immunotherapy effective against low-immunogenic breast cancer while reducing side effects. Cell Rep (2022) 40(8):111256. doi: 10.1016/j.celrep.2022.111256 36001966

[114] Bolte LA Lee KA Björk JR Leeming ER Campmans-Kuijpers MJE de Haan JJ. Association of a mediterranean diet with outcomes for patients treated with immune checkpoint blockade for advanced melanoma. JAMA Oncol (2023) 9(5):705–9. doi: 10.1001/jamaoncol.2022.7753 PMC9936383 36795408

[115] Galus Ł Michalak M Lorenz M Stoińska-Swiniarek R Tusień Małecka D Galus A. Vitamin D supplementation increases objective response rate and prolongs progression-free time in patients with advanced melanoma undergoing anti–PD-1 therapy. Cancer (2023) 129(13):2047–55. doi: 10.1002/cncr.34718 37089083

[116] Ferrere G Tidjani Alou M Liu P Goubet A-G Fidelle M Kepp O. Ketogenic diet and ketone bodies enhance the anticancer effects of PD-1 blockade. JCI Insight (2021) 6(2):e145207. doi: 10.1172/jci.insight.145207 33320838 PMC7934884

[117] Messaoudene M Pidgeon R Richard C Ponce M Diop K Benlaifaoui M. A natural polyphenol exerts antitumor activity and circumvents anti–PD-1 resistance through effects on the gut microbiota. Cancer Discov (2022) 12(4):1070–87. doi: 10.1158/2159-8290.CD-21-0808 PMC9394387 35031549

[118] Morehead LC Garg S Wallis KF Siegel ER Tackett AJ Miousse IR. Increased response to immune checkpoint inhibitors with dietary methionine restriction. bioRxiv (2023). doi: 10.2139/ssrn.4329452 PMC10526448 37760436

[119] Lévi F. Daytime versus evening infusions of immune checkpoint inhibitors. Lancet Oncol (2021) 22(12):1648–50. doi: 10.1016/S1470-2045(21)00607-0 34780710

[120] Qian DC Kleber T Brammer B Xu KM Switchenko JM Janopaul-Naylor JR. Effect of immunotherapy time-of-day infusion on overall survival among patients with advanced melanoma in the USA (MEMOIR): a propensity score-matched analysis of a single-centre, longitudinal study. Lancet Oncol (2021) 22(12):1777–86. doi: 10.1016/S1470-2045(21)00546-5 34780711

[121] Dizman N Govindarajan A Zengin ZB Meza L Tripathi N Sayegh N. Association between time-of-day of immune checkpoint blockade administration and outcomes in metastatic renal cell carcinoma. Clin Genitourin Cancer (2023) 21(5):530–6. doi: 10.1016/j.clgc.2023.06.004 37495481

[122] Nomura M Hosokai T Tamaoki M Yokoyama A Matsumoto S Muto M. Timing of the infusion of nivolumab for patients with recurrent or metastatic squamous cell carcinoma of the esophagus influences its efficacy. Esophagus (2023) 20(4):722–31. doi: 10.1007/s10388-023-01006-y PMC10123478 37093536

[123] Rousseau A Tagliamento M Auclin E Aldea M Frelaut M Levy A. Clinical outcomes by infusion timing of immune checkpoint inhibitors in patients with advanced non-small cell lung cancer. Eur J Cancer (2023) 182:107–14. doi: 10.1016/j.ejca.2023.01.007 36758475

[124] Yeung C Kartolo A Tong J Hopman W Baetz T. Association of circadian timing of initial infusions of immune checkpoint inhibitors with survival in advanced melanoma. Immunotherapy (2023) 15(11):819–26. doi: 10.2217/imt-2022-0139 37191006

[125] Cortellini A Barrichello APC Alessi JV Ricciuti B Vaz VR Newsom-Davis T. A multicentre study of pembrolizumab time-of-day infusion patterns and clinical outcomes in non-small-cell lung cancer: too soon to promote morning infusions. Ann Oncol (2022) 33(11):1202–4. doi: 10.1016/j.annonc.2022.07.1851 35953005

[126] Gonçalves L Gonçalves D Esteban-Casanelles T Barroso T Soares de Pinho I Lopes-Brás R. Immunotherapy around the clock: impact of infusion timing on stage IV melanoma outcomes. Cells (2023) 12(16):2068. doi: 10.3390/cells12162068 37626878 PMC10453728

[127] Ballesta A Innominato PF Dallmann R Rand DA Lévi FA. Systems chronotherapeutics. Pharmacol Rev (2017) 69(2):161–99. doi: 10.1124/pr.116.013441 PMC5394920 28351863

[128] Sulli G Lam MTY Panda S. Interplay between circadian clock and cancer: new frontiers for cancer treatment. Trends Cancer (2019) 5(8):475–94. doi: 10.1016/j.trecan.2019.07.002 PMC7120250 31421905

[129] Khan M Du K Ai M Wang B Lin J Ren A. PD-L1 expression as biomarker of efficacy of PD-1/PD-L1 checkpoint inhibitors in metastatic triple negative breast cancer: A systematic review and meta-analysis. Front Immunol (2023) 14:1060308. doi: 10.3389/fimmu.2023.1060308 36949944 PMC10027008

[130] Lopes S Pabst L Dory A Klotz M Gourieux B Michel B. Do proton pump inhibitors alter the response to immune checkpoint inhibitors in cancer patients? A Meta-analysis. Front Immunol (2023) 14:1070076. doi: 10.3389/fimmu.2023.1070076 36776847 PMC9910608

[131] Shuboni D Yan L. Nighttime dim light exposure alters the responses of the circadian system. Neuroscience (2010) 170(4):1172–8. doi: 10.1016/j.neuroscience.2010.08.009 20705120

[132] Cajochen C Jud C Münch M Kobialka S Wirz-Justice A Albrecht U. Evening exposure to blue light stimulates the expression of the clock gene PER2 in humans. Eur J Neurosci (2006) 23(4):1082–6. doi: 10.1111/j.1460-9568.2006.04613.x 16519674

[133] Mohawk JA Green CB Takahashi JS. Central and peripheral circadian clocks in mammals. Annu Rev Neurosci (2012) 35:445–62. doi: 10.1146/annurev-neuro-060909-153128 PMC3710582 22483041

[134] Durrant J Michaelides EB Rupasinghe T Tull D Green MP Jones TM. Constant illumination reduces circulating melatonin and impairs immune function in the cricket Teleogryllus commodus. PeerJ (2015) 3:e1075. doi: 10.7717/peerj.1075 26339535 PMC4558066

[135] Wei L Yue F Xing L Wu S Shi Y Li J. Constant light exposure alters gut microbiota and promotes the progression of steatohepatitis in high fat diet rats. Front Microbiol (2020) 11:1975. doi: 10.3389/fmicb.2020.01975 32973715 PMC7472380

[136] Dallmann R Brown SA Gachon F. Chronopharmacology: new insights and therapeutic implications. Annu Rev Pharmacol Toxicol (2014) 54(1):339–61. doi: 10.1146/annurev-pharmtox-011613-135923 PMC3885389 24160700

[137] Sulli G Manoogian ENC Taub PR Panda S. Training the circadian clock, clocking the drugs, and drugging the clock to prevent, manage, and treat chronic diseases. Trends Pharmacol Sci (2018) 39(9):812–27. doi: 10.1016/j.tips.2018.07.003 PMC7249726 30060890

[138] Lee KA Thomas AM Bolte LA Björk JR de Ruijter LK Armanini F. Cross-cohort gut microbiome associations with immune checkpoint inhibitor response in advanced melanoma. Nat Med (2022) 28(3):535–44. doi: 10.1038/s41591-022-01695-5 PMC8938272 35228751

[139] Kostopoulou V Katsouyanni K. The truth-telling issue and changes in lifestyle in patients with cancer. J Med Ethics (2006) 32(12):693. doi: 10.1136/jme.2005.015487 17145907 PMC2563347

[140] Paepke D Wiedeck C Hapfelmeier A Kiechle M Brambs C. Lifestyle modifications after the diagnosis of gynecological cancer. BMC Womens Health (2021) 21(1):260. doi: 10.1186/s12905-021-01391-5 34182983 PMC8240378

